# Intimate partner violence and help-seeking: The role of femicide news^☆^

**DOI:** 10.1016/j.jhealeco.2022.102722

**Published:** 2023-01

**Authors:** Marco Colagrossi, Claudio Deiana, Davide Dragone, Andrea Geraci, Ludovica Giua, Elisa Iori

**Affiliations:** a European Commission, Joint Research Centre (JRC), Italy; b University of Cagliari, Italy; c University of Bologna, Italy; d University of Pavia, Italy

**Keywords:** Gender-based violence, Helpline, Intimate partner violence, Physical violence, Psychological violence, Sexual violence

## Abstract

Exploiting high-frequency data from the Italian anti-violence helpline, police reports of domestic abuse and maltreatments, and a unique geolocalized dataset on killings of women, we show that the news coverage of a femicide triggers an increase in help-seeking behavior. The effect is detectable in the period following the news and in the province where the femicide has occurred. Additionally, help-seeking increases more when the general interest and news coverage are higher. These findings are consistent with a model in which femicide news increase expectations about future intimate partner violence in case no action is taken. Our results imply that recurrent information campaigns and public discussion can foster help-seeking from survivors of gender-based violence.

## 1 Introduction

Intimate partner violence (IPV) is one of the most frequent forms of violence against women. One in three women worldwide has suffered from physical and/or sexual violence in her lifetime (WHO, 2021); one in five women who have had at least one partner has experienced physical or sexual violence by an intimate partner (European Union Agency for Fundamental Rights, 2015). The most extreme act of violence against women is the gender-related murder of a woman by a man, or *femicide*. This is not an uncommon phenomenon: in 2017, 87,000 women in the world were intentionally killed, and in 58% of the cases the perpetrator was a current or former partner or a family member (Stöckl et al., 2013, UNODC, 2018). To fight against IPV, more than 150 countries have taken initiatives and implemented policy interventions, including stricter sanctions for the abusing partners, faster police and judiciary procedures, financing of shelter homes, communication and education campaigns (WHO, 2012, WHO, 2013).^1^

Recognition and reporting of IPV is a fundamental step to end gender-based violence (Heise and Garcia-Moreno, 2002). In this paper, we focus on this step and study the determinants of help-seeking behavior by survivors of intimate partner violence. We consider Italy and we investigate the effect of femicide news on the calls to the Italian helpline against gender-based violence, and on the reporting of domestic abuse and maltreatments to the police.

The Italian case is instructive because figures on IPV are in line with the international evidence. About 31% of women aged 16 to 70 have suffered from physical or sexual violence at least once in their life (ISTAT, 2021c); in 2018, 80% of femicides were made by a current or former partner, or by a family member (55% and 25%, respectively, ISTAT, 2020). The Italian policies to fight against gender-based violence, protect the survivors, and prosecute the offenders, are aligned to those implemented worldwide and recommended by the WHO. Among them, there is the creation of the dedicated *1522* helpline. Available 24/7 in several languages, the *1522* helpline is the official, public-funded service that provides support and information to survivors of violence and stalking, and guidance to public and private social-health and support services on the national territory, including “anti-violence” centers and shelters (ISTAT, 2019).

The effect of a femicide news on a survivor of IPV is in principle ambiguous. On the one hand, it can increase the salience of the expected benefits of seeking for help (or, equivalently, of the expected costs of not taking action) due to, e.g., empathy or identification with the victim. On the other hand, the femicide news can increase fear of reprisal by the violent partner, especially if calling for help does not immediately stop violence. To empirically study which effect dominates, we combine different sources covering the period 2015–2019: (i) a unique, geolocalized, high-frequency dataset of killings of girls and women, (ii) a novel administrative dataset on province-level weekly calls to the *1522* helpline, (iii) an administrative database of province-level, monthly police reports on domestic abuse and maltreatments, (iv) a dataset on national weekly Google searches of femicide victims, and (v) data extracted from the Global Database of Events, Language and Tone, on broadcast, print, and web daily news related to violence against women.

Three main results emerge. First, helpline calls increase by 0.054 calls per 100,000 inhabitants in a given week after a femicide is reported in the news. This effect, which corresponds to a 11% increase, is detectable in the province and in the week after the femicide has occurred. Second, monthly police reports increase by 0.118 per 100,000 inhabitants, which corresponds to a 6% rise. Third, we show that not all femicide news have the same effects on help-seeking behavior. Calls to the helpline increase more when the femicide spurs more general interest, as captured by Google searches for the victim’s name, when news coverage on gender-related violence is more intense, when the victim of the femicide is young, and when the method of killing is brutal.

The findings of this paper add to a growing literature on IPV and the effectiveness of policy interventions aimed at reducing violence against women. For example, Aizer and Dal Bo (2009) show that the introduction of no-drop laws leads to increased help-seeking behavior, and Chin and Cunningham (2019) show that laws allowing for the officer discretion to arrest are associated with a lower number of intimate partner homicides. Amuedo-Dorantes and Deza (2022) report that sanctuary policies that limit the co-operation of law enforcement agencies with federal immigration authorities, induce a decrease in femicides among Hispanic women in the US. Iyer et al. (2012) find that higher female representation in the local governments is associated to a rise in help-seeking by survivors of gender-based crimes, and Miller and Segal (2019) document higher rates of IPV reporting when the female representation among police officers increases. Levy and Mattsson (2020) and Gauthier (2022) show that the reporting of gender-based violence and abuse has increased following the #MeToo movement, which is consistent with the idea that heightened public awareness boosts the credibility of reporting, and decreases the uncertainty about the consequences and fear of retaliation (Lee and Suen, 2020, Cheng and Hsiaw, 2022). Similarly, Colagrossi et al. (2022) estimate increased reporting of violence following a media campaign advertising the use of the Italian anti-violence helpline. Our empirical results show that documenting and talking about femicides and gender-related violence is important to raise recognition and reporting of IPV. The finding that more media coverage and higher general interest induce more help-seeking behavior, provides empirical support to policy interventions aimed at raising and maintaining awareness about IPV.

This paper also contributes to understand the drivers of help-seeking behavior and of gender-based violence, which have been recently investigated by, e.g., Yilmaz, 2018, Tur-Prats, 2019, González and Rodríguez-Planas, 2020, Angelucci and Heath, 2020, and Tur-Prats (2021). By focusing on the drivers of the IPV survivors’ response, we provide a complementary perspective to the literature studying crime and the impact of exposure to the media, which typically focuses on the offenders’ response.^2^ Card and Dahl (2011) exploit results of football games broadcast and show a rise in IPV violence after an upset loss, while Jensen and Oster (2009) find that the introduction of cable television in India is associated to significant decreases in the acceptability of IPV. Dahl and DellaVigna (2009) find that violent crime decreases on days with larger theater audiences for violent movies. Gulino and Masera (2022) estimate that news of corruption scandals affect the propensity of supermarket customers to steal. As in our case, the effect of the news is localized in time and space. Our paper adds to this literature by showing that help-seeking behavior from victims of IPV is a specific response to a femicide news — and not to any news about non-IPV related murders. If the victim is young or the femicide is brutal, calls increase more. Murder-suicides, instead, reduce the impact of the news. Factors such as emotional impact for the general public and the IPV survivors, salience and coverage of the news, and increased awareness and information are possible drivers for the increase in help-seeking behavior after a femicide news is released.

Our results translate into important policy implications. Raising awareness on violence against women and covering femicide events in the news can play a valuable role in prompting survivors to report violence. The response to news salience in terms of help-seeking behavior, however, is short-lived. Policy makers may remedy by promoting frequent and systematic information campaigns and by encouraging public discussion about gender-based violence. The finding that both helpline calls and police reports increase following the news of a femicide also suggests that potential help-seekers may be effectively reached not only via established channels (i.e. police) but also via dedicated services such as helplines.

The remainder of this paper is structured as follows: Section 2 describes the data used for the analysis and provides background information on femicides, helpline calls and police reporting in Italy. Section 3 proposes a model of help-seeking behavior, while Section 4 outlines our empirical approach. Section 5 describes our findings. Sections 6, 7 discuss the results and conclude.

## 2 Data and descriptive statistics

We focus on Italy and combine five data sources with information on (i) femicides, (ii) calls to the *1522* helpline, (iii) police reports for domestic abuse and maltreatments, (iv) Google searches for the names of femicide victims, and (v) news coverage about violence against women. See Table A.1 for an overview and summary statistics.

The first source of information comes from a unique, geolocalized, high-frequency dataset on killings of women compiled by the Italian non-governmental organization *Casa delle Donne per Non Subire Violenza*, which provides support and shelter for women who undergo violence. The database records all murders of girls and women reported by the local and national press in Italy. It includes information on the date and the place in which the murder occurred, the motivation, the type of weapon used, the relationship between the survivor and the offender, and personal characteristics such as age, job occupation, history of violence, illness and use of substances.^3^

The second source is an administrative database on calls to the *1522* helpline over the years 2015–2019, released by the Department for Equal Opportunities. The helpline is sponsored by the Italian Government and is the official contact point for information about anti-violence centers (AVCs) and shelters.^4^ Helpline operators are specifically trained to deal with victims of violence, and they can redirect calls to the police, hospitals or health centers upon request or in case of emergency. To reduce under-reporting by the IPV survivors, callers’ anonymity is always guaranteed. The dataset consists of weekly *counts* at the province level of the total number of calls and of calls made by victims of violence. The total number of calls refers to any call made to seek help, report a violence, or request information on any support service addressing violence or stalking. The calls flagged as *made by victims* are a subset of the total calls. These are calls for which callers explicitly declare that they have experienced some form of violence and provide basic socio-demographic information to helpline operators. In all cases, only valid calls are considered, i.e. they must be congruous with the services provided by the helpline or linked to its purpose. This means that nuisance calls and those made by mistake are excluded. To preserve anonymity, no further individual information on the caller is disclosed.

Third, we use a novel administrative database provided by the Department of Public Security of the Italian Ministry of the Interior. Drawn from the Italian Police archive of criminal records and charges, the dataset contains monthly counts of police reports on domestic abuse and maltreatments, disaggregated at province level over the period 2015–2019.

The fourth database is based on Google searches for the name of each victim of femicide occurred over the years 2015–2019. This allows us to measure the general interest or curiosity for specific femicides at the national weekly level.

The fifth dataset is constructed using the Global Database of Events, Language and Tone (GDELT), an open big data platform of meta-information extracted from broadcast, print, and web news collected worldwide. By retrieving all items with the tags ‘gender violence’, ‘rape’, ‘youth and gender based violence’, ‘domestic violence’, ‘rape and sexual violence’, we construct a national measure of the daily share of news on gender-related violence over the years 2016–2019.^5^

Finally, data on population, homicides, poverty and education used in the robustness exercises come from the official Census statistics released by the Italian Institute of Statistics (ISTAT).

### 2.1 Femicides in Italy

**Fig. A.1:**
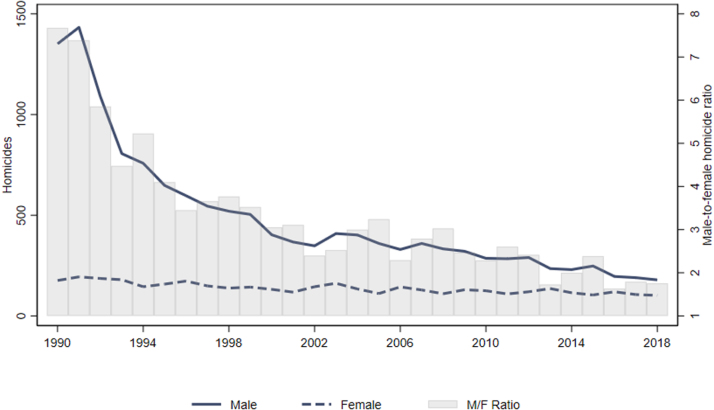
Number of homicides in Italy. *Note*: Solid and dashed lines represent the yearly total number of men and women killed, respectively. Bars indicate the male-to-female homicide ratio.

Italy has one of the lowest homicide rates globally, yet the male-to-female homicide ratio has been steadily decreasing over time.^6^ The decreasing trend in homicides has been fully driven by a reduction in male homicides, as female homicides have remained constant over time (Fig. A.1). In the official statistics, of the 345 homicides that occurred in Italy in 2018, 212 were male victims and 133 were female victims (ISTAT, 2019). On average, one woman is killed every three days.

**Fig. 1:**
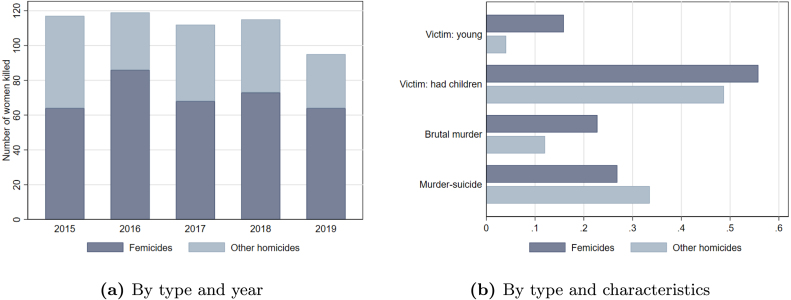
Female homicides in Italy. *Note*: Dark bars indicate femicides; light bars refer to other female homicides due to, e.g., depression, health reasons or disability of the victim or her relatives. Panel (a) reports the total number of female homicides by year. Panel (b) reports the averages of the victims’ characteristics: (i) young (below 28 years old); (ii) with children; (iii) brutal method (beating, hitting, pushing and strangulation); (iv) murder-suicides.

Our dataset on femicides contains rich information which is unavailable in the official statistics. Based on the murder’s motive, we can distinguish between “femicides” and “other female homicides”. The former category includes gender-based murders where the woman was killed for reasons associated with a sentimental or sexual relationship.^7^ The latter category includes all female homicides where the motive is related to the health conditions of the murderer, the victim, or her relatives; they are typically due to the existence of a severe disability, terminal illnesses or depression, and they often involve elderly women (87% of these victims are over 60 years old). A few events in this latter group consist of murders for which the motive is unknown and the killer is not a former or current partner, or due to unrelated motives (e.g., money).

Women victims of femicide are murdered mostly by their current or former partners (82%) or by other family members (9%). In the remaining 9% of the cases, the perpetrator is an acquaintance (such as a colleague or a client). Considering other female homicides in our sample, a third of which involves a murder-suicide, women are either killed by their partner (71%) or by a family member (29%, typically their adult child).

Panel (a) of Fig. 1 shows that female homicides are typically more than 100 per year and that on average three out of four are femicides; panel (b) of Fig. 1 offers an overview of the victims’ characteristics. The share of murder-suicides is lower among femicides, where the victim is typically younger and is more likely to be killed with brutal and violent means, such as beating, hitting, pushing from heights and strangulation.^8^

**Fig. 2:**
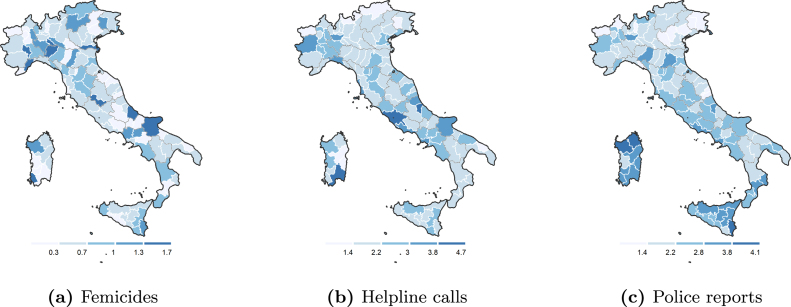
Geographical distribution of femicides, helpline calls and police reports in Italy. *Note*: The legends report (a) the total number of femicides per 100,000 inhabitants, (b) the average monthly number of calls per 100,000 inhabitants and (c) the average monthly number of police reports for episodes of domestic abuse and maltreatments per 100,000 inhabitants in the period 2015–2019 for each province. Gray and white lines refer to regional (NUTS-2) and province (NUTS-3) boundaries, respectively.

**Fig. A.2:**
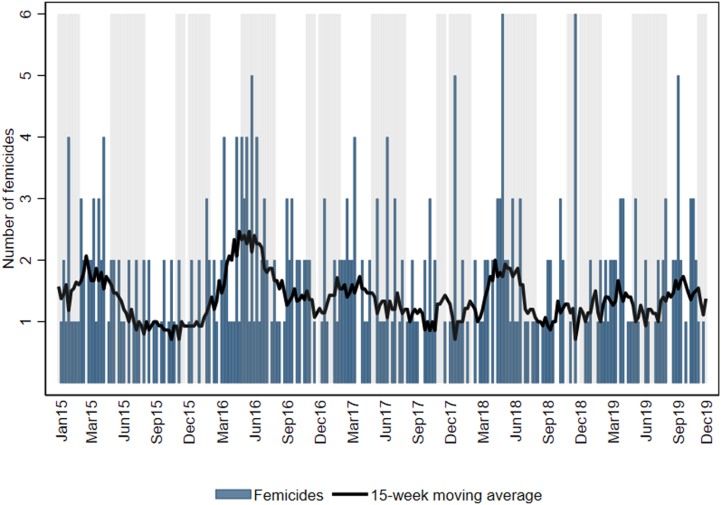
Femicides over the years 2015–2019. *Note*: Number of femicides: weekly count (blue histograms) and 15-week moving average (black line). The light gray backgrounds mark the winter (Dec–Feb) and summer (Jun–Aug) seasons. (For interpretation of the references to color in this figure legend, the reader is referred to the web version of this article.)

Fig. A.2 describes the timeline of femicides. On average, 1.36 femicides occur weekly. While no noticeable time pattern is present, rates of violence seem to be highest in the summer months. Panel (a) of Fig. 2 presents the geographical distribution of the average weekly number of femicides per 100,000 inhabitants by province. Although there exist differences across the 110 Italian provinces and the 20 regions, no clear geographical pattern emerges. In particular, there is no compelling evidence of the typical North–South divide that exists when considering average income, female employment, education, alcohol consumption, and life expectancy.

### 2.2 Seeking for help in Italy: helpline calls and police reports

To measure help-seeking behavior, we consider calls to the *1522* helpline and police reports for domestic abuse and maltreatments. Both pathways can be freely accessed by IPV survivors, but they differ in the way they operate. The *1522* helpline offers support to victims of violence and stalking, and acts as the first contact point for anti-violence centers (AVCs). This was the case for three out of four calls received in 2019 (ISTAT, 2021a). Police reporting, instead, initiates a judiciary investigation which can eventually lead to the incapacitation of the offender.

Reporting to the police does not guarantee that violence will end in the short-run, as the offender is able to return home before the judicial sentence.^9^ Calling the helpline can be a safer and more effective method to escape violence, as shelters and support measures can be immediately activated while the survivor’s privacy is protected. When violence is reported to the police, on the other hand, the abuser is notified and this may increase the risk of retaliation on the victim. Around one in three victims who call the helpline do not report to the police or drop charges due to fear of the abuser, not having anywhere safe to go or because the police advised not to report (ISTAT, 2021c). Because of this, the two paths are not perfect substitutes, and help-seekers typically report to the police once they have found refuge in a safe place. Note that the action of the helpline focuses on the survivor, while the police and judiciary interventions act on the offender.

In our analysis, we use the weekly province-level counts of the total number of calls to the helpline and of those classified specifically as made by victims of violence.^10^ On average, the *1522* helpline receives more than 20,000 calls every year. As shown in Table 1 for the year 2019, with more than 93% of the calls directly made “for oneself”, the helpline effectively achieves its goal of being a reference for individuals seeking help, support and information. Specifically, about 50% of callers seek information on anti-violence centers or on the services provided by the helpline and about 30% of the calls consist of requests for help. When the calls report violence (about 5% of the overall calls), in 2 cases out of 3 they are made by the victims’ relatives (parents, siblings, or children) or friends.^11^ As reported by ISTAT using aggregated 2019 data, the offender is usually a current or former partner (79% of cases) or a family member (13%); in 92% of the cases, calls refer to instances of repeated violence (ISTAT, 2021c).^12^

**Table 1:** Calls to the *1522* helpline in 2019.

Reason for calling		Call made for oneself	Call made by family or friends	Call made by professionals
Request for information	10,816 (50.8%)	95%	1%	4%
Request for help: violence	6483 (30.5%)	100%	0%	0%
Reporting violence	1098 (5.2%)	29%	67%	4%
Request for help: stalking	897 (4.2%)	100%	0%	0%
Emergency	174 (0.8%)	100%	0%	0%
Other	1822 (8.6%)	100%	0%	0%
Total	21,290 (100%)			

*Note*: *Request for information* includes information about national anti-violence centers and the helpline, legal information, information about procedures from professionals and information about the legal responsibilities of public operators; *Other* includes reporting of malfunctioning services, misleading media coverage and calls out of target.

**Fig. A.3:**
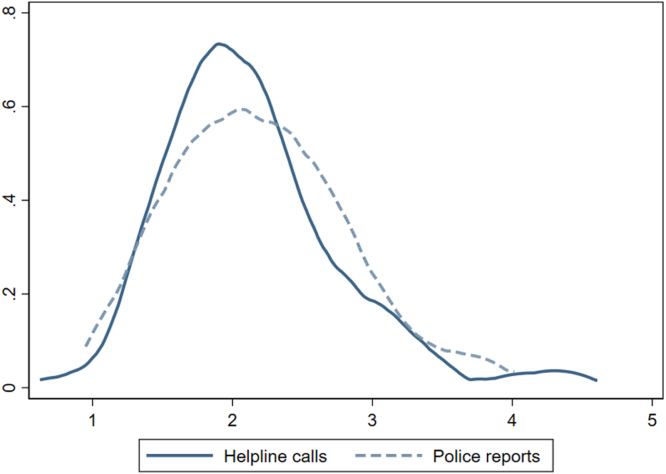
Distribution of helpline calls and police reports. *Note*: Monthly average number of helpline calls (solid line) and police reports for domestic abuse and maltreatments (dashed line), rescaled by 100,000 inhabitants, years 2015–2019.

The data on police reports contains the monthly *counts* of province-level reports for domestic abuse and maltreatments in the period 2015–2019. Every year police reports for this type of crimes are almost 21,000. As shown in Fig. A.3, helpline calls and police reports have very similar distributions.

**Fig. 3:**
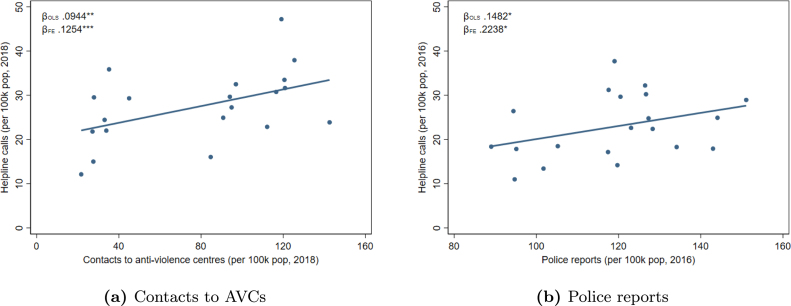
Helpline calls, contacts to AVCs and police reports. *Note*: Correlations between the number of helpline calls and (a) the number of people who contacted an Anti-Violence Center (AVC) in 2018 and (b) the number of police reports for crimes related to domestic violence in 2016. Each dot corresponds to a regional value. $\beta_{OLS}$ refers to a simple correlation, $\beta_{FE}$ includes southern region fixed effects. For each variable we consider the latest year available.

ISTAT.

Fig. 3 shows that helpline calls positively correlate with both contacts to anti-violence centers (panel a) and police reports for crimes associated to domestic violence (panel b).^13^ An increase of one helpline call per 100,000 inhabitants is associated to a rise in both contacts to AVCs and police reports by 0.13 and 0.22, respectively. Although this is just a correlation, it suggests that helpline calls and police reports capture different, but strongly associated, aspects of the same phenomenon.

Panels (b) and (c) of Fig. 2 map the geographical distribution of the average monthly number of calls and the average monthly number of reports for domestic abuse and maltreatments by province.^14^ Although there exist some differences in the use of the helpline service and in police reporting, both across and within regions, no clear spatial clusters emerge.

### 2.3 General interest and news coverage measures

**Fig. A.4:**
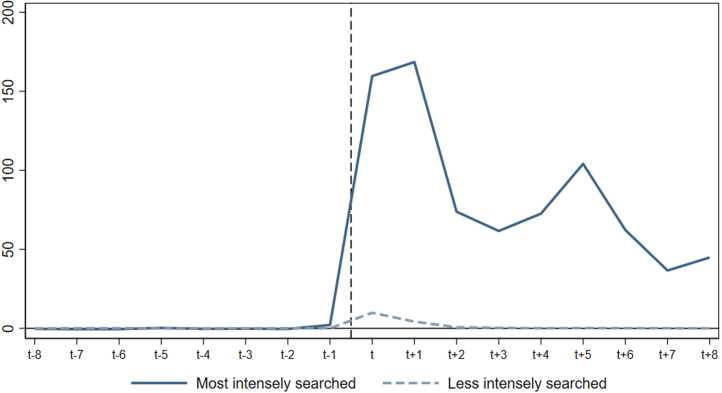
Google Search intensity on victims’ names. *Note*: The solid dark line is the average Google Search index for the most intensely searched names (i.e. above the fourth quartile of the overall distribution). The dashed light line refers to the average search index of less intensely searched names (i.e. below the fourth quartile of the overall distribution). All series are recentered around the date of each victim’s murder (week t).

To measure the interest of the general public raised by each femicide event, we use Google Search data. For each victim’s name we construct a time series of weekly Google searches at the national level. We label as *Most Searched* the events for which total searches within the eight weeks following femicide date are in the highest quartile. Fig. A.4 shows the average *Most Searched* time series (dark solid line) against the less searched ones (light dashed line). The *Most Searched* time series increase when the femicide news are released, reach the peak in the following week, and then fade away over time.

**Fig. 4:**
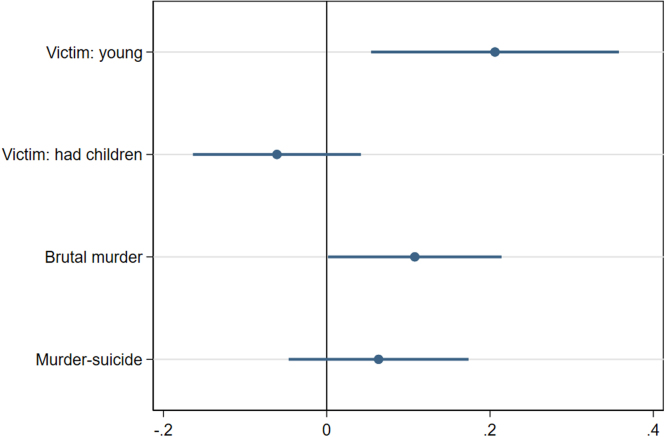
Most searched femicides and victims’ characteristics. Note: OLS estimates (blue dots) and robust 95% confidence intervals associated to the probability that a femicide is flagged as most searched on the web.

Fig. 4 shows the correlation between the dummy variables identifying most searched femicides, the characteristics of the victims and the methods of killing. The events generating more interest are associated with the victims being young. If anything, victims with children are less intensely searched. Brutal murders (i.e. murders by strangulation, pushing from heights, beating, and hitting) are positively correlated with the most searched femicides. Murder-suicides are not.

**Fig. 5:**
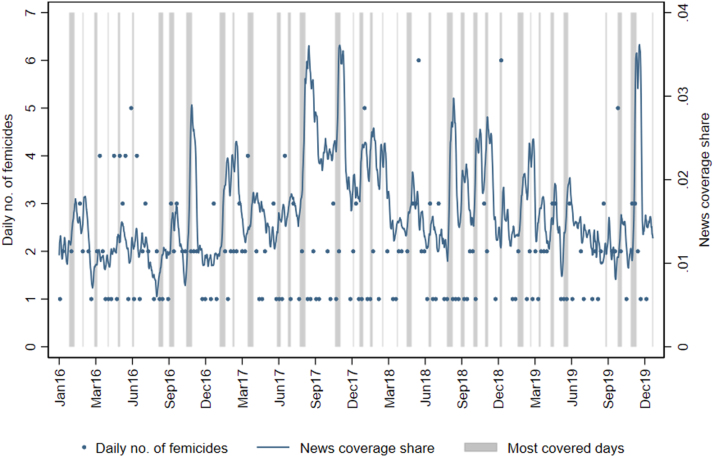
News coverage of gender-based violence and femicides. *Note*: The solid line represents the share of news covering topics related to gender-based violence and femicides in any given day, computed as a 15-day moving average. Each dot is the number of femicides occurred in each week. The vertical bars indicate periods of high news coverage.

Data from GDELT consists of the daily share of news mentioning topics related to gender violence. This index proxies for the intensity of the news coverage of gender-based violence and femicides events at the national level. Over the period considered, the index is never zero, which implies that there is always some coverage of gender-related violence in the news. Coverage, however, is quite heterogeneous over time, as shown by the solid line of Fig. 5. On average, 1.6% of the news cover topics related to gender-based violence every day. Some, but not all peaks, correspond to the International Women’s Day (March 8) and the International Day for the Elimination of Violence against Women (November 25). The news coverage share based on GDELT and Google searches are positively and significantly correlated with an elasticity of 0.55, which suggests that both capture public discussion about femicides.

We consider days where coverage increases significantly with respect to the previous two weeks and label as *Most Covered* the femicides that occur in those days. There are 37 periods of increasing coverage (gray bars, about 20% of the 2016–2019 period), which span from 2 to 14 days. The *Most Covered* femicides are 35% of all femicides in our database.

## 3 Taking action against intimate partner violence: A model

In industrialized countries, two main patterns of IPV emerge: a severe and escalating form of violence characterized by multiple forms of abuse, and a more moderate form of violence which occasionally erupts into physical aggression (Johnson, 1995, Johnson and Ferraro, 2000). In response to IPV, “some women resist, others flee, while still others attempt to keep the peace by giving in to their husbands’ demands” (Heise and Garcia-Moreno, 2002 p.95). In general, most abused women seem to adopt strategies that maximize their safety and that of their children. Factors such as fear of retribution by the abusive partner, lack of economic means, concern for the children, and hope that the man will change, may prevent an IPV survivor from taking active actions to end violence (Heise and Garcia-Moreno, 2002).

Based on these considerations, in this section we present a simple model to describe the trade-offs affecting the choice of taking action against IPV.^15^

**Fig. 6:**
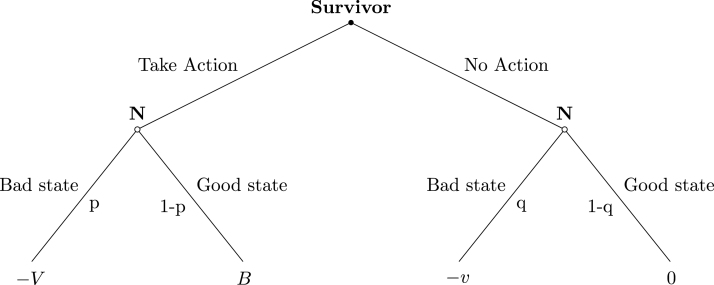
Taking action against violence. *Notes*: V denotes the level of violence as retaliation and B the benefits of interrupting violence as a consequence of taking action; v denotes the expectation of violence – which can depend on past violence and other factors μ – if no action is taken.

Consider the choice between two options: not taking action against violence, or taking action by, e.g., calling the helpline or reporting to the police. After the choice is made, a good or a bad state can realize. Survivor i expects the bad state to occur with subjective probability p if action is taken, and with subjective probability q if no action is taken (see Fig. 6).^16^

If an action is taken, in the good state the IPV survivor obtains B≥0, whose magnitude depends on, e.g., the net benefits of psychological counseling and the possibility of escaping violence altogether. These benefits are not only individual-specific, but they also depend on targeted policies implemented at both the national and the local level, such as the availability of helplines and shelters. In the bad state, the IPV survivor obtains −V<0 due to, e.g., additional violence as retaliation by the abusive partner. In the following we will refer to B as the benefit of taking action, and to V as the level of violence as retaliation.

When no action is taken, in the good state the survivor receives zero. This possibility describes a situation in which violence is reduced, or even ceases, even if the survivor does not take any action.^17^ If this is not the case, taking no action yields −v<0 in the bad state, a situation in which violence persists. To take into account the existence of different patterns of violence, let $v=v_{i,t}$ and assume that, at time t, survivor i’s expected level of violence depends on past violence and other factors, according to

(1) $$v_{i,t}=\rho v_{i,t-1}+\mu_{i,t}$$

The term ρ≥0 describes the persistence of past violence $v_{i,t-1}$. The term $\mu_{i,t}$ represents the role of factors unrelated to the individual experience of past violence, and that can affect the trajectory of violence in case no action is taken. These factors can include, e.g., social norms (González and Rodríguez-Planas, 2020, Colagrossi et al., 2022), employment shocks (Aizer, 2010), other shocks (Card and Dahl, 2011) or, as we study in this paper, news about a femicide. According to Eq. (1), histories of moderate violence which occasionally erupt into bursts of physical aggression correspond to $\rho \in \left(0,1\right)$ and $\mu_{i,t}>0$. Histories of escalating violence are instead characterized by ρ>1.^18^

The IPV survivor observes the level of past violence $v_{i,t-1}$ and forms expectations about the consequences of taking action. Under risk neutrality, the expected utility associated to the two options is:

$$U\left(\text{Action}\right)=\left(1-p_{i,t}\right)B_{i,t}-p_{i,t}V_{i,t};\;\;U\left(\text{No Action}\right)=-q\left(\rho v_{i,t-1}+\mu_{i,t}\right)$$

By comparing the two expected utilities, the following holds:

Proposition 1 *Survivors of intimate partner violence take action if the expectation of new violence is large enough,*

(2) $$\mu_{i,t}>{\overset{ˆ}{\mu }}_{i,t},$$

*where*

$${\overset{ˆ}{\mu }}_{i,t}=\frac{p_{i,t}}{q_{i,t}}V_{i,t}-\frac{1-p_{i,t}}{q_{i,t}}B_{i,t}-\rho v_{i,t-1}$$

If the threshold ${\overset{ˆ}{\mu }}_{i,t}$ is negative or zero, any expectation (or threat) of new violence is sufficient to trigger an action. More in general, and consistent with the empirical research on IPV and the factors that affect the decision to leave an abusive partner, taking action against violence is more likely to occur when expected violence as retaliation ($p_{i,t}V_{i,t}$) is low, and when the expected benefits of escaping violence by taking action ($\left(1-p_{i,t}\right)B_{i,t}$) and the persistence of violence ($\rho v_{i,t-1}$) are large.^19^

Remark 1 Taking action against violence is more likely when

• The net benefits B of taking action are large,
• The level of violence V as retaliation is small,
• The probability p of violence as retaliation is small,
• The probability q of violence in case of inaction is large,
• Expected violence is along an explosive trajectory.

To understand the effect of a femicide news on a survivor of IPV, consider the case in which the femicide news $n_{i,t}$ an individual receives, increases (i) the expectation of new violence if no action is taken and (ii) the probability of violence as retaliation if action is taken, i.e. $\mu^{\prime }\equiv \frac{\partial \mu_{i,t}}{\partial n_{i,t}}\geq 0$ and $p^{\prime }\equiv \frac{\partial p_{i,t}}{\partial n_{i,t}}\geq 0$.^20^ In such a case, taking action is more likely after a femicide news if

(3) $$\mu^{\prime }>\frac{B_{i,t}+V_{i,t}}{q_{i,t}}p^{\prime }$$

Expression (3) describes two opposite effects that make the response to a femicide news ambiguous. The first one relies on the upward revision of expectations of future violence ($\mu^{\prime }$) due to, e.g., identification with the victim, the realization that (the cost of) future violence is higher than previously expected, or the expectation that the femicide news will trigger emulation by the abusive partner. The second effect is due to fear that taking action will trigger additional violence. This is a realistic possibility, especially when calling for help or reporting to the police does not immediately stop violence by the abusive partner. While the former effect implies that femicide news induce more IPV survivors to take action, the latter deters it. Hence:

Proposition 2 *After a femicide is reported in the news, more IPV survivors take action if the motives driven by increased expectations of new violence in case no action is taken, dominate the increased expectation of violence as retaliation in case action is taken.*

## 4 Empirical strategy

We adopt a regression difference-in-differences setting where the treatment of interest is the release of a femicide news occurred in province p in week t. This event-study design can be formalized as follows (Athey and Imbens, 2022):

(4) $$Y_{pt}=\alpha +\overset{-1}{\underset{\pi =-4}{\sum }}\beta_{\pi }D_{pt+\pi }+\overset{4}{\underset{\tau =1}{\sum }}\beta_{\tau }D_{pt+\tau }+\delta_{t}+\gamma_{p}+\epsilon_{pt}.$$

**Fig. A.5:**
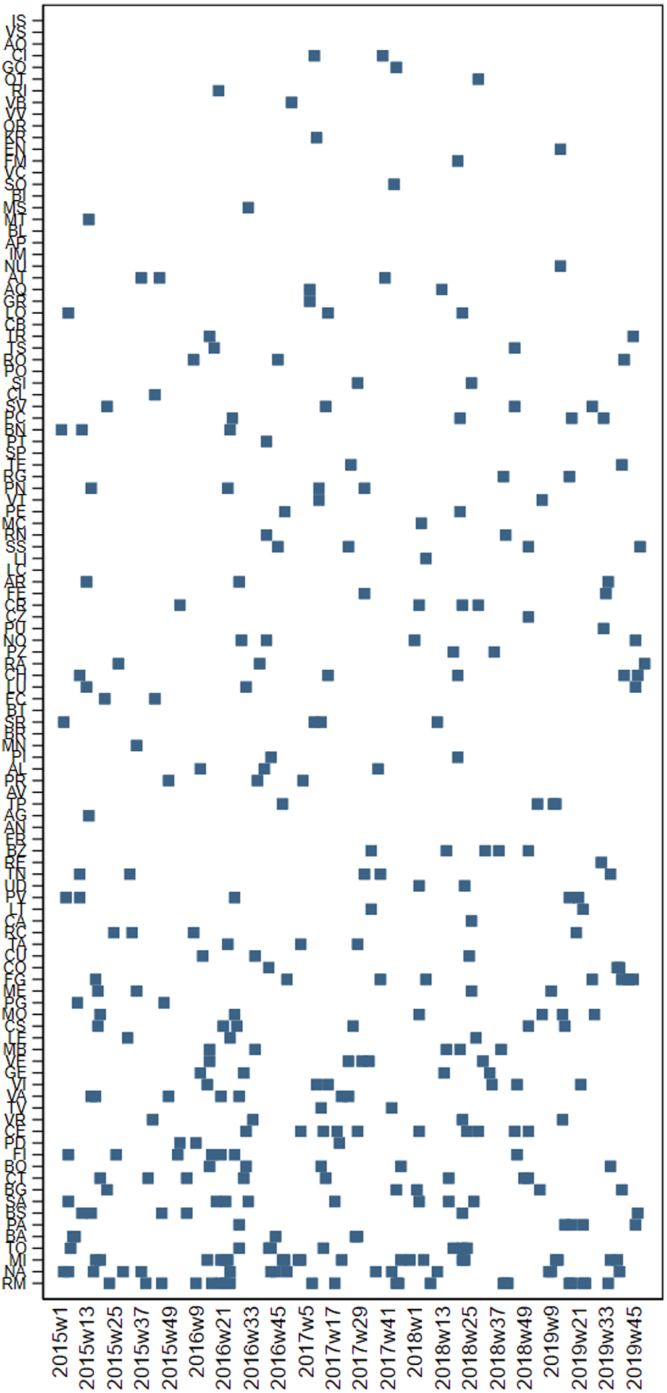
Distribution of femicides across provinces and weeks. *Note*: Spatial–temporal distribution of the treatment. Each square represents a femicide in a given province in a given week (w). The provinces are ordered by population size.

The outcome $Y_{pt}$ is the number of calls to the *1522* helpline per 100,000 resident population measured in province p in week t. The dummies $D_{pt+\pi }$ and $D_{pt+\tau }$ describe 4 pre- and 4 post-treatment periods associated to each femicide. Fig. A.5 displays the time and geographical variation of our treatment, where provinces are ordered by population size. As expected, femicides occur more frequently in the more populated areas, such as in the provinces of Rome (RM), Naples (NA) and Milan (MI).

Given that there may be repeated events in a given province p, we consider a 16-week window around each event. Thus, the dummies associated to π=−4 and τ=4 include all periods prior to t−4 (up to t−8) and those after t+4 (up to t+8), respectively.^21^ The terms $\delta_{t}$ and $\gamma_{p}$ allow for week and province fixed effects, respectively, while $\epsilon_{pt}$ is the error term. All regressions are weighted by the 2011 population at province level. Throughout the analysis, model (4) is estimated using standard errors clustered at province level, with the week before a femicide occurs, i.e. week t−1, as baseline time period.

We study whether our results are robust to the possible existence of geographical spillovers across provinces by checking the Stable Unit Treatment Value Assumption (SUTVA). To alleviate concerns related to spurious correlations, we perform a placebo test using female homicides not flagged as femicides.

Further exercises, discussed and reported in Appendix B, are: an additional falsification test based on future femicide events; supplementary analyses including province-specific linear differential trends in pre-existing levels of violence, poverty and education, and province-year and week-year-region fixed effects. We also test the robustness to a specification without population weights and the use of a Poisson pseudo-log-likelihood (PPML) model. Last, to address possible concerns about the comparability of treatment and control units, we re-run the analysis following the procedure recently proposed by Imai et al. (2021).^22^

Police reports on domestic abuse and maltreatments per 100,000 resident population measured in province p in month m are used as a second outcome. In this case, we adapt Eq. (4) in order to exploit the different time variability, i.e. months instead of weeks.^23^

The exercises above shed light on the effect of the femicide news on help-seeking behavior. To understand the effect of different types of femicides we consider the victims’ demographics (age and the presence of children), the brutality of the murder (death by strangulation, pushing from heights, beating, and hitting) and murder-suicides. Finally, we study whether *Most Searched* and *Most Covered* femicides reinforce the effect of news on help-seeking.

## 5 Results

**Fig. 7:**
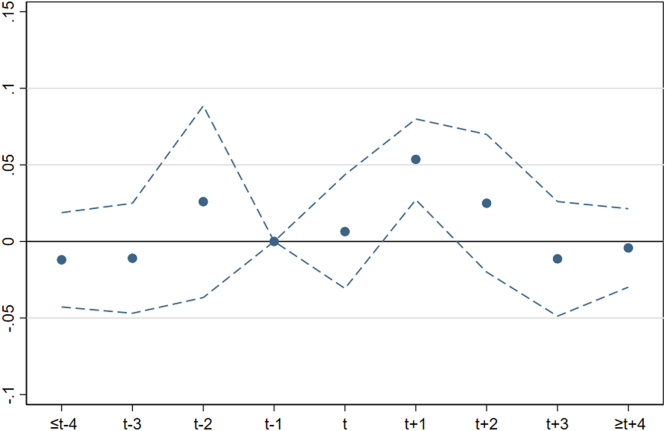
Effect of femicide news on helpline calls, weakly data. Note: OLS estimates (dots) of lags and leads and corresponding 95% confidence intervals (dashed lines) as from Eq. (4). The baseline period is t−1 (one week prior to the occurrence of a femicide). The dependent variable is the number of helpline calls per 100,000 population. Estimates include province and week-year fixed effects. Standard errors are clustered at province level.

Fig. 7 shows the effect of the release of news about the occurrence of a femicide on the overall number of helpline calls per 100,000 inhabitants. The estimated coefficients, also reported in column 1 of Table A.2 imply an increase in helpline calls by 0.054 calls per 100,000 inhabitants in the week following the occurrence of a femicide, i.e. at t+1. Given an average value of 0.492 calls per 100,000 population, this corresponds to a 11% increase. The effect is positive and statistically significant, but it is short-lived, as the effect appears at t+1 and fades afterwards. Reassuringly, coefficients reveal no significant differences across treated and control provinces in the periods prior to the femicide, suggesting that the parallel trend assumption holds.

We obtain equivalent results if we consider the number of calls from victims only, as shown in column 2 of Table A.2. Here, the coefficient associated to the week following the femicide (t+1) is halved. However, given that the average number of calls per 100,000 population made by victims of violence is 0.218, the estimated increase amounts to 11%, similarly to the previous specification. This result is especially important because it confirms we are correctly measuring the outcome of interest, i.e. we can confidently exclude that the effect on the overall number of calls is driven by third-party callers or by those who have not actually experienced violence.^24^

**Fig. A.6:**
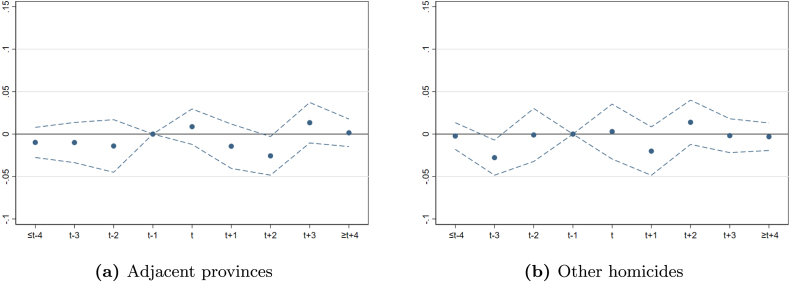
Effect of femicide news on helpline calls, additional falsification exercises. *Note*: OLS estimates of lags and leads and corresponding 95% confidence intervals as from Eq. (4). The baseline time period is t−1 (one week prior to the occurrence of a femicide in a given province). The dependent variable is the number of helpline calls per 100,000 population. Treatment is assigned to (a) adjacent provinces, or (b) female homicides associated to reasons other than gender-based violence. Population-weighted estimates include province and week-year fixed effects. Standard errors are clustered at province level.

Then, we test the geographical reach of the effect of a femicide news on salience about IPV and help-seeking behavior by including in the treatment group the provinces adjacent to the treated units. From an econometric point of view, this is relevant because, if there are geographical spillovers, the Stable Unit Treatment Value Assumption (SUTVA) does not hold and the estimated effects are likely to be downward biased. The results reported in panel (a) of Fig. A.6 excludes the existence of any spillover effect of the femicide news over the adjacent provinces.^25^ We can therefore conclude that the effect of femicide news on help-seeking behavior is positive, statistically significant, and local in space and time.

As the crime literature highlights, criminal statistics may suffer from under-reporting (see, e.g., MacDonald, 2002). Our analysis aims at understanding whether the news about femicides may decrease under-reporting of IPV. Our results suggest this is the case. However, in our context, measurement error due to mis-reporting may be an issue. The difference-in-differences estimates will be unbiased if such mis-reporting occurs systematically in both treated and control units. It might be possible, of course, that after hearing about a femicide some survivors contact directly the police or local NGOs without calling the helpline. In such a case, calls counts would understate the total number of people seeking help in any given week. If this was more likely in treated provinces, our estimates may be biased towards zero. Nevertheless, we find an almost identical elasticity to the femicide news when using a different source of data, i.e. police reports, which alleviates this possible concern. There could also be a downward bias if, following the femicide news, some survivors in non-treated provinces call more for help. However, this issue appears to be negligible, as Fig. A.6 supports the absence of spillovers across provinces. On the contrary, we might estimate an upward bias if femicide news systematically shift reporting practices of helpline operators or police in case of help-seekers located in treated provinces. This is unlikely to occur within such a narrow time window (i.e. the week or month following the news) and because resources allocation is managed at the national level.

As a placebo, we substitute our main treatment variable with a dummy taking value one when the murder of a woman is not flagged as femicide. This is the case when the motive is unknown or ascribable to health reasons, namely when the offender is gravely depressed or when either the survivor, her children or her partner-killer suffer from terminal illnesses or severe disability. We do not expect this type of news to affect the propensity to call the anti-violence helpline because these murders are typically associated to situations of extreme disadvantage and distress, and are generally portrayed as unrelated to gender-based violence. Consistent with this hypothesis, columns 3 and 4 of Table A.2 show that the coefficients associated to this specification are never statistically different from zero, both when analyzing the overall number of calls and those made by victims of violence only (see also Panel (b) of Fig. A.6).

Our results are robust to the inclusion of province-specific linear trends based on past violence, poverty, and literacy, week-region-year fixed effects specifications. They are also robust to using a Poisson pseudo log-likelihood model, instead of the linear model in Eq. (4). To address possible concerns about treated units switching in and out of the treatment, we then consider the alternative difference-in-differences design proposed by Imai et al. (2021). The obtained results are in line with our main empirical exercise. An additional placebo exercise based on future femicides lends further support to our empirical strategy and results. Results from these robustness checks and the alternative specifications are reported in Appendix B.

**Fig. 8:**
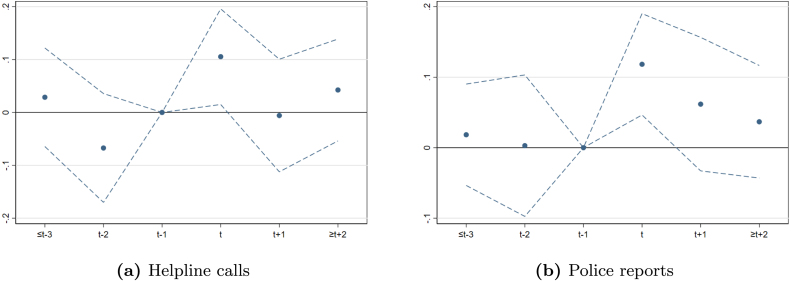
Effect of femicide news on helpline calls and police reports, monthly data. *Note*: OLS estimates (dots) of lags and leads and corresponding 95% confidence intervals (dashed lines) as from Eq. (4). The baseline period is t−1 (one month prior to the occurrence of a femicide). The dependent variables are (a) the number of police reports for domestic abuse and maltreatments, and (b) the number of helpline calls per 100,000 population. Estimates include province and month-year fixed effects. Standard errors are clustered at province level.

Fig. 8 shows the effect of the release of news about the occurrence of a femicide on helpline calls and police reports on domestic abuse and maltreatments per 100,000 inhabitants, using monthly data. The results, reported in columns 1 and 3 of Table A.3, are in line with the ones obtained using weekly data. In particular, the estimated coefficients associated to the month following the occurrence of a femicide (t+1) imply an increase in helpline calls and police reports by 0.105 and 0.118, respectively. This corresponds to a 5% increase in the month after the femicide. Again, the effect is positive and statistically significant, but short-lived. As shown in column 2 of Table A.3, the conclusions are similar when considering the sub-sample of helpline calls made by victims only.

**Table 2:** Effect of femicide news on helpline calls, by type of femicide.

Treatment	(1)	(2)	(3)	(4)	(5)	(6)
	Most	Most	Victim		Brutal	Murder-
	covered	searched	Young	Children	femicide	suicide
Baseline: The effect of the news of a femicide						
t − 4 or earlier	0.004	−0.003	−0.011	−0.018	−0.010	−0.013
	(0.015)	(0.012)	(0.014)	(0.013)	(0.016)	(0.016)
t − 3	−0.035	−0.002	−0.009	0.006	−0.025	−0.008
	(0.025)	(0.026)	(0.021)	(0.019)	(0.028)	(0.020)
t − 2	0.022	0.024	0.022	0.012	0.032	0.030
	(0.026)	(0.021)	(0.031)	(0.038)	(0.028)	(0.036)
**t**	0.001	0.006	−0.004	0.014	0.017	0.010
	(0.021)	(0.019)	(0.021)	(0.024)	(0.023)	(0.022)
**t**+**1**	0.035*	0.047*	0.036*	0.078***	0.028*	0.079***
	(0.018)	(0.025)	(0.020)	(0.020)	(0.015)	(0.016)
**t**+**2**	0.015	0.037	0.003	0.067*	0.019	0.015
	(0.016)	(0.023)	(0.031)	(0.035)	(0.024)	(0.026)
**t**+**3**	−0.013	−0.022	−0.027	0.011	−0.018	−0.008
	(0.024)	(0.017)	(0.017)	(0.026)	(0.024)	(0.020)
**t**+**4or later**	−0.002	0.003	−0.010	0.015	−0.015	−0.002
	(0.013)	(0.014)	(0.017)	(0.013)	(0.022)	(0.013)

Differential effect of the selected treatment						

t − 4 or earlier	−0.022	−0.005	−0.001	0.012	−0.004	0.004
	(0.015)	(0.022)	(0.015)	(0.030)	(0.018)	(0.019)
t − 3	0.004	−0.027	−0.004	−0.029	0.036	−0.009
	(0.020)	(0.024)	(0.038)	(0.024)	(0.045)	(0.036)
t − 2	0.022	0.050	0.025	0.025	−0.018	−0.015
	(0.051)	(0.052)	(0.029)	(0.023)	(0.071)	(0.031)
**t**	0.021	0.044	0.053	−0.014	−0.026	−0.013
	(0.028)	(0.040)	(0.038)	(0.028)	(0.023)	(0.024)
**t**+**1**	0.054*	0.048*	0.089	−0.044	0.067**	−0.091**
	(0.030)	(0.026)	(0.057)	(0.031)	(0.029)	(0.036)
**t**+**2**	0.039	−0.006	0.103*	−0.074**	0.012	0.039
	(0.035)	(0.027)	(0.059)	(0.035)	(0.033)	(0.045)
**t**+**3**	−0.006	0.031	0.067	−0.037	0.012	−0.011
	(0.023)	(0.035)	(0.042)	(0.023)	(0.032)	(0.029)
**t**+**4or later**	0.001	−0.013	0.018	−0.031	0.028	−0.007
	(0.012)	(0.021)	(0.025)	(0.022)	(0.028)	(0.016)

Observations	28,231	28,231	28,231	28,231	28,231	28,231
R-squared	0.307	0.307	0.307	0.307	0.307	0.307

*Note*: The baseline period is t−1 (one week prior to the event of interest). The dependent variable is the number of helpline calls per 100,000 population. Population-weighted estimates include province and week-year fixed effects. Young victims are aged 28 or less. Beating/hitting includes any type of beating, hitting, pushing from heights and strangling. Standard errors are clustered at province level. *** p < 0.01, ** p < 0.05, * p < 0.1.

It seems intuitive to conjecture that not all femicide news have the same impact on help-seeking behavior. Given our data, we can test this conjecture and study: (i) the role of news coverage and general interest, (ii) the potential impact of the individual characteristics of the victim, and (iii) the possible effect of specific features of the murder. These exercises implicitly test whether the treatment “news of a femicide” is not homogeneous across treated units.

Table 2 shows the empirical results. First, we find that *Most Covered* femicides are associated to additional 0.054 calls per 100,000 inhabitants (column 1).^26^ Second, *Most Searched* femicides, i.e. those that have raised more Google searches for the victims’ names, are associated to relatively more calls (column 2). Note that the baseline coefficients associated to the least searched events are similar to the overall results. The most searched events, instead, are associated to a further increase in the number of helpline calls by 0.048 calls per 100,000 inhabitants (around 9% with respect to the sample mean). Third, femicides in which the victim is young and childless are associated to more calls (columns 3 and 4). Fourth, most brutal femicides are associated to more calls (14%, column 5). Murder-suicide deaths, instead, are associated to lower rates of helpline calls by 19% (column 6).^27^

## 6 Discussion

The findings of the empirical analysis can be summarized as follows:

Result 1 *Femicide news increase help-seeking behavior.*

Result 2 *Calls to the helpline increase more when: (i) the victim is young and childless, (ii) the femicide is brutal, (iii) the femicide raises more interest, (iv) the news coverage is higher.*

Based on the theoretical model, the increase in helpline calls and police reports is consistent with the hypothesis that femicide news increase future expectations of violence by women suffering from IPV, and, in particular, that the motives driven by increased expectations of new violence in case no action is taken overcome the increased expectation of violence as retaliation in case action is taken.

The increase in both helpline calls and police reports shows that help-seeking behavior is responsive to femicide news. This is especially true considering that IPV survivors in a given area are likely to be at different stages of their path of escaping violence when the news of a femicide are released. For instance, there will be IPV survivors who are at an early stage of the process (e.g., gathering information via the helpline – thus, the increase in calls), but also IPV survivors who are in a later stage (e.g., people who have left their abuser already and report to the police – hence, the increase in police reports).^28^

Result 1 shows that disclosing femicide news can contribute to the recognition of violence and the reporting by IPV survivors, which is considered in the literature and in the WHO guidelines as the first step to end gender-based violence (Heise and Garcia-Moreno, 2002).

We estimate that femicide news increase helpline calls by 11% in the week after the event is reported and police reports increase by 6% in the following month. These magnitudes are compatible with the literature studying factors that affect IPV reporting. For instance, Miller and Segal (2019) estimate that in the US a 7.4 percentage point increase in the share of female officers among police (i.e. the average increase over the period 1979–1990) boost reporting of domestic violence by 13.6 percentage points (around 25% of the sample mean). In the context of the effect of the #MeToo movement on reporting of sex crimes, Levy and Mattsson (2020) estimate a 10% increase in sex crime reports over a large sample of OECD countries. Using a sample of US cities, Gauthier (2022) finds that #MeToo accounts for approximately 25% of the increase in sex crime reporting over the period 2017–2019. In the context of Italy, Colagrossi et al. (2022) evaluate the impact of a special anti-violence campaign launched in 2020 a few weeks after the first Covid lockdown. By disentangling the effect of the campaign from the one of the lockdown, they find that helpline calls double in the first week after the launch of the campaign and increase by 300% in the fifth week. In their case, however, the magnitude of the effect is plausibly enhanced by the overlap with very stringent and prolonged lockdown measures, which most likely made the campaign reach an otherwise impracticable audience size. Finally, Gulino and Masera (2022) explore the impact of news on (non-violent) crime-related outcomes in Italy and find that local corruption scandals increase the probability of stealing in supermarkets by 16% in the few days after the news. Similar to our results, the effect is localized in space and time.

Result 1 could be due to different, non mutually exclusive, reasons. For example, a survivor can be triggered to take action because she shares a history of IPV similar to the victim. Such response could be more likely when there are shared demographic characteristics, or specific elements of the femicide that act on the individual sensitivity of the IPV survivor, such as the age of the victim, or the brutality of the murder.

The choice of taking action could also respond to the salience of the news for the survivor, which is likely affected by how many times, and how intensively, the IPV survivor is exposed to the femicide news. News that circulate more in the media, or are more present in the public debate and in informal discussions between family, friends and acquaintances, can therefore induce a higher likelihood of taking action. Higher news coverage can not only increase the salience of the femicide for the survivor, but also reduce the possible stigma associated to calling for help.

A related possibility is that the femicide news and its coverage conveys information that is useful *per se* for the victim to better assess the expected consequences of taking action. Cheng and Hsiaw (2022) for example, show that people can be reluctant to report misconduct when there are information frictions. If the reporting of femicide news reduces information frictions about, e.g., the typical trajectories of violence, the consequences of inaction, or the availability of shelters (which in the theoretical model correspond to ρ, μ and B), then we would expect more help-seeking behavior. It is also possible that the femicide news indirectly trigger help-seeking behavior by increasing awareness by family members and friends, who may call the helpline or convince the survivor to do so.

Disentangling each channel is not possible with our data. Since the characteristic of those who call the helpline or report to the police are not available, we cannot directly test whether the choice of taking action is responsive to shared characteristics between the victim and the survivor. However, our second set of results provides some hints about the role of the media coverage and the characteristics of the femicides that can further trigger help-seeking behavior.

We show that more news coverage of femicides is associated to more calls to the helpline. This finding provides empirical support to policy interventions currently implemented worldwide and aimed at increasing awareness, providing information and informing about the routes for ending gender-based violence (Colagrossi et al., 2022). Importantly, our results show no evidence of saturation effects whereby the IPV victims do not respond anymore to the news. In other words, taking the current media coverage as the reference, our results show no evidence of satiation, which supports the policy implication that, for fighting violence against women, “the more news, the better”.

Google searches measure the interest for the general population, which proxies the number of times and the intensity of exposition of the IPV survivor to the femicide news. Femicides that trigger more interest induce more calls to the helpline. Differently from the proxy for news coverage, which only contains meta-data and does not allow measuring how often victims are mentioned in the news, Google search data allows matching search intensity to each victim’s names. The fact that these two exercises speak to each other further corroborates our conclusions about the role of enhanced information and awareness.

Interestingly, not all femicides have the same effect on help-seeking. Femicides where the victim is young or childless induce a stronger response. This is consistent with the salience or identification hypothesis presented above, and with the 2014 ISTAT’s survey on the prevalence of violence in Italy, which reports that the share of women having suffered violence in the past 12 months is highest among those aged 16–24 years old (11.6%, compared to an average of 4.5% among those aged 16–70).^29^ Conversely, the absence of children is likely to be related to age effects, also in view of the fact that Italy is the EU country with the highest age at first birth, at 31.3 years old.^30^

Analogously, when the femicide is brutal, calls to the helpline increase more. Again, the salience hypothesis seems a reasonable explanation, as it is likely that the exceptional nature of the event grabs more attention and, possibly, increases the salience of the IPV survivor’s current situation.

As shown in Section 2, these types of femicides are significantly correlated to Google Searches. It is therefore possible that they raise more interest and salience in the general population, as well as in the IPV survivor, which in turn increases help-seeking behavior. On the contrary, murder-suicides reduce the impact of the femicide news. This category of femicides does not further increase the propensity of the IPV survivor to call for help, possibly because they are often the result of a single, non-brutal violent act, and not a sequence of repeated aggressions. It is possible that they are perceived by IPV survivors as less relatable to their personal experience.

## 7 Conclusions

Violence against women is a violation of human rights (Council of Europe, 2011) and a public health concern. Not only it produces consequences on the survivors’ physical and mental health, but it also hampers female autonomy and self-realization. Femicides are the extreme realization of a much more common phenomenon of violence perpetrated against women.

In this paper, we shed light on the help-seeking behavior of survivors of intimate partner violence (IPV). We study how victims of IPV react to the news of a femicide by estimating whether they trigger more calls to a dedicated helpline and more reporting to the police. Our theoretical model points to two opposite mechanisms. On the one hand, the news might foster help-seeking by increasing the victims’ expectations about future violence if no action is taken, or by raising the expected benefit associated with help-seeking. On the other hand, reaching out for help and support might be discouraged if femicide news increase the fear of retaliation by the violent offender.

To empirically test the effect of the news, we adopt a difference-in-differences design to estimate the response of IPV survivors seeking for help after a femicide is reported in the news. We use unique data sources on femicide events in the years 2015–2019, calls to the Italian anti-violence helpline, police reports, Google Searches and news coverage of gender-related violence.

We find that after a femicide is reported in the news, calls to the helpline increase by 11%. The effect appears to be short-lived, and geographically localized. Consistently, police reports increase by 6%. We show that calls increase more when the victim is young and childless, the femicide is brutal, raises more interest and curiosity, and it is more covered in the news. Overall, these findings provide empirical support to the relevance of media coverage as an important channel to fight violence against women.

The main policy implication of our findings is that talking about femicides not only raises awareness, but it is also an important trigger for help-seeking behavior by the survivors of intimate partner violence. This is commonly recognized as an important step to end intimate partner violence, and our paper provides empirical support to the impact of information campaigns and the increased importance given in the news to femicides. The salience of the news and its impact on help-seeking behavior quickly fades away. To avoid this, the policy maker should promote recurrent information campaigns and public discussion around gender-based violence, together with active local support services for survivors aimed at increasing the expected benefits of calling for help.

## Declaration of Competing Interest

The authors declare that they have no known competing financial interests or personal relationships that could have appeared to influence the work reported in this paper.

## Figures and Tables

Italian Cause of Death database, ISTAT (1990–2018).

**Table A.1:** Data used in the analysis and descriptive statistics.

Data	Aggregation	Time unit	Mean	Std.dev.	Min	Max
**Female homicides**	Municipality	Day				
Femicides			0.012	0.115	0.000	3.000
Other homicides			0.007	0.086	0.000	2.000
**Helpline calls**	Province	Week				
All valid calls			0.492	0.481	0.000	5.731
Calls from victims only			0.218	0.280	0.000	4.585
**Police reports**	Province	Month				
Reports for domestic abuse and maltreatments			2.192	1.181	0.000	9.013
**Google searches**	National	Week				
Google search index			49.754	180.107	−3.289	2081.630
Most Searched			0.270	0.444	0.000	1.000
**News coverage**	National	Day				
News coverage share			0.016	0.009	0.002	0.096
Most Covered			0.203	0.403	0.000	1.000

*Note*: Helpline calls and police reports are per 100,000 population. Sources are Casa delle donne per non subire violenza NGO (Female homicides), Department of Equal Opportunities (Helpline calls), Department of Public Security (Police reports), Google (Google searches), GDELT (News coverage). All data cover the years 2015–2019, except GDELT (2016–2019).

**Table A.2:** Effect of female homicides news on helpline calls.

Treatment	(1)	(2)	(3)	(4)
	Femicides		Other homicides	
Dep. variable	Total calls per 100k pop	Calls from victims per 100k pop	Total calls per 100k pop	Calls from victims per 100k pop
t − 4 or earlier	−0.012	−0.004	−0.003	−0.002
	(0.016)	(0.005)	(0.016)	(0.008)
t − 3	−0.011	0.012	−0.031	−0.028***
	(0.018)	(0.010)	(0.024)	(0.011)
t − 2	0.026	0.025	0.049	−0.001
	(0.032)	(0.017)	(0.041)	(0.016)
**t**	0.006	0.000	0.014	0.003
	(0.019)	(0.013)	(0.031)	(0.017)
**t**+**1**	0.054***	0.025***	−0.012	−0.020
	(0.013)	(0.009)	(0.022)	(0.015)
**t**+**2**	0.025	0.021	0.027	0.014
	(0.023)	(0.016)	(0.024)	(0.013)
**t**+**3**	−0.011	−0.001	0.026	−0.002
	(0.019)	(0.009)	(0.026)	(0.010)
**t**+**4or later**	−0.004	−0.006	−0.005	−0.003
	(0.013)	(0.008)	(0.017)	(0.008)

Observations	28,231	28,231	28,231	28,231
R-squared	0.307	0.184	0.306	0.184

*Note*: The baseline period is t−1 (one week prior to the event of interest). The dependent variable is the number of helpline calls (total or from victims only) per 100,000 population. Population-weighted estimates include province and week-year fixed effects. Standard errors are clustered at province level. *** p < 0.01, ** p < 0.05, * p < 0.1.

**Table A.3:** Effect of femicide news on helpline calls and police reports, monthly data.

Dep.	(1)	(2)	(3)
variable	Total calls per 100k pop	Calls from victims per 100k pop	Police reports per 100k pop
t − 3 or earlier	0.029	−0.008	0.018
	(0.048)	(0.020)	(0.037)
t − 2	−0.067	−0.020	0.003
	(0.053)	(0.031)	(0.051)
**t**	0.105**	0.084***	0.118***
	(0.046)	(0.030)	(0.037)
**t**+**1**	−0.006	−0.023	0.062
	(0.054)	(0.039)	(0.048)
**t**+**2or later**	0.042	0.044*	0.037
	(0.049)	(0.026)	(0.041)

Observations	6360	6360	6360
R-squared	0.554	0.438	0.532

*Note*: The baseline period is t−1 (one month prior to the event of interest). The dependent variable is the number of helpline calls (total or from victims only) per 100,000 population and the number of police reports for domestic abuse and maltreatments per 100,000 population. Population-weighted estimates include province and month-year fixed effects. Standard errors are clustered at province level. *** p < 0.01, ** p < 0.05, * p < 0.1.

**Table B.1:** Effect of femicide news on helpline calls, robustness tests.

	(1)	(2)	(3)	(4)	(5)	(6)
t − 4 or earlier	−0.014	−0.014	−0.014	0.002	−0.008	−0.007
	(0.014)	(0.015)	(0.015)	(0.011)	(0.013)	(0.026)
t − 3	−0.013	−0.014	−0.014	0.009	0.002	0.009
	(0.017)	(0.018)	(0.018)	(0.021)	(0.022)	(0.042)
t − 2	0.024	0.023	0.023	0.044	0.024	0.049
	(0.032)	(0.032)	(0.032)	(0.027)	(0.025)	(0.046)
**t**	0.004	0.003	0.003	0.008	−0.015	−0.023
	(0.018)	(0.018)	(0.018)	(0.020)	(0.022)	(0.042)
**t**+**1**	0.052***	0.050***	0.050***	0.059***	0.081***	0.137***
	(0.014)	(0.014)	(0.014)	(0.020)	(0.025)	(0.041)
**t**+**2**	0.024	0.022	0.022	0.042*	−0.008	−0.013
	(0.023)	(0.023)	(0.023)	(0.023)	(0.024)	(0.046)
**t**+**3**	−0.012	−0.014	−0.014	−0.035	−0.029	−0.050
	(0.019)	(0.019)	(0.020)	(0.022)	(0.018)	(0.036)
**t**+**4or later**	−0.008	−0.009	−0.009	−0.005	−0.016	−0.028
	(0.013)	(0.013)	(0.014)	(0.013)	(0.013)	(0.026)

Observations	28,231	28,231	28,231	27,972	28,231	28,231
R-squared	0.311	0.319	0.319	0.464	0.165	
Population-weighted	✓	✓	✓	✓		
Violence linear trends	✓					
Poverty linear trends		✓				
Education linear trends			✓			
Province-Year FE				✓		
Week-Year-Region FE				✓		
Estimator	OLS	OLS	OLS	OLS	OLS	PPML

*Note*: The baseline period is t−1 (one week prior to the event of interest). The dependent variable is the number of calls to the *1522* helpline per 100,000 population. Population-weighted estimates include province and week-year fixed effects. Columns 1 to 3: province-specific linear trends based on the total number of female homicides (violence), the incidence of households in potential economic distress (poverty) and the incidence of illiterates (education). Column 4 includes province-year and week-year-region fixed effects. Column 5 reports unweighted results. In column 6 the model is estimated using the Poisson’s pseudo log-likelihood model (PPML). Standard errors are clustered at province level. *** p < 0.01, ** p < 0.05, * p < 0.1.

**Fig. B.1:**
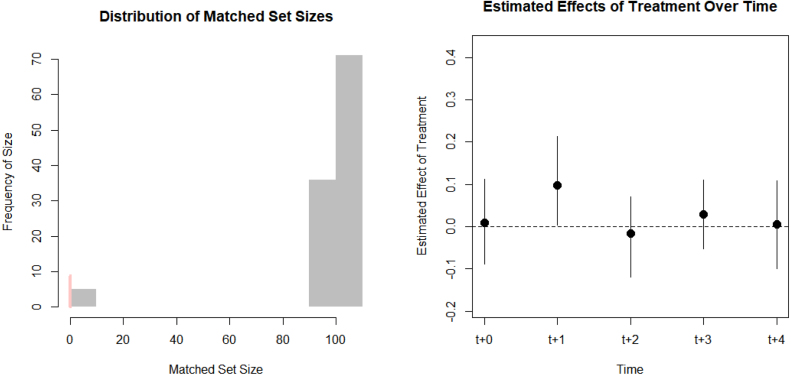
Effect of femicide news on helpline calls, alternative procedure. *Note*: Procedure proposed by Imai et al. (2021) and implemented via the *PanelMatch* R package. The left plot shows the number of matched control units that share the same treatment history as a treated observation. The red thin bar at zero represents the number of treated observations that have no matched control units. The right plot displays coefficients and corresponding 95% confidence intervals estimated based on a matching method that adjusts for the treatment and covariate histories during the four weeks period prior to the treatment.

**Fig. B.2:**
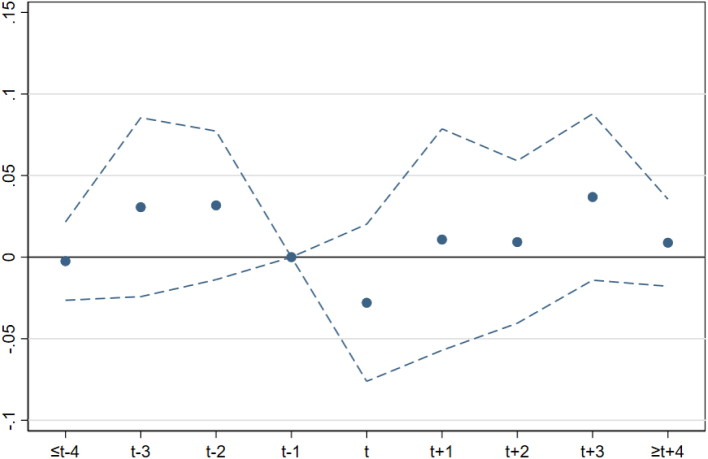
Effect of femicide news on helpline calls, future femicides. *Note*: OLS estimates of lags and leads and corresponding 95% confidence intervals as from Eq. (4). The baseline time period is t−1 (one week prior to the occurrence of a femicide in a given province). The dependent variable is the number of helpline calls per 100,000 population. Treatment is assigned to future femicides in the same province. Population-weighted estimates include province and week-year fixed effects. Standard errors are clustered at province level.

## Appendix A Figures and tables

See Fig. A.1, Fig. A.2, Fig. A.3, Fig. A.4, Fig. A.5, Fig. A.6 and Table A.3, Table A.1, Table A.2.

## Appendix B Robustness checks and placebo exercises

A possible endogeneity concern is that, irrespective of the femicide event and its media coverage, the occurrence of a femicide can depend on the local prevalence of violence. If this is the case, a simple OLS would deliver biased estimates. The high frequency of our data helps controlling for both spatial heterogeneity and time shocks by including province and week-year fixed effects. This allows to exploit the within-province-week variation in the data.

Moreover, our identification strategy is grounded on the assumption that provinces where femicides occur, and provinces that do not experience such events follow a parallel trend in the pre-treatment period, i.e. in the weeks prior to the femicide. If this is the case, we can, to some extent, trust that in these areas femicides are not endogenously related to differential trends of violence. Empirically, the parallel trend assumption is satisfied if the lead coefficients $\beta_{\pi }$ are not statistically different from zero, which implies that the treated provinces are trending similarly to the untreated ones before the femicide. Under the above assumptions, the lag coefficients $\beta_{\pi }$ measure the effect of a femicide news on helpline calls in the weeks following the event.

Nonetheless, other local characteristics might still be potentially correlated with the prevalence of violence and help-seeking behavior from survivors. As robustness checks, in Table B.1, columns 1 to 3, we report the results of additional regressions in which we add province-specific linear differential trends in pre-existing levels of violence, poverty, and education, respectively.^31^ The inclusion of these controls is meant to absorb additional possible confounding factors, but it does not change our main conclusions. Similar results are shown in column 4, where we include province-year and week-region fixed effects to exploit the residual province-week-year variation within the same region.^32^ These fixed effects take into consideration potentially important confounding factors, such as changes in regulations enacted by regional entities and public funding to anti-violence centers (AVCs). The results, however, are consistent with our main conclusions. Controlling for this additional time-varying heterogeneity, we detect a positive and statistical significant impact on helpline calls also at t+2.

In column 5 of Table B.1 we also show that our results are not sensitive to the use of population weights. In addition, we address potential incidental parameters issues, by estimating a Poisson’s pseudo log-likelihood (PPML) model, which accounts for dependent variables with many zeros, different patterns of heteroskedasticity, and is robust to outcome measurement errors (Santos Silva and Tenreyro, 2006). As shown in column 6, all estimated coefficients of the robustness checks are in line with the main results.

A notable feature of our setup is that the same unit can be treated more than once over time. As shown in Fig. A.5, for a non-negligible number of provinces we do observe more than one femicide in the time horizon considered. To avoid this feature to bias our results and following the recent developments in the literature, we apply the approach proposed by Imai et al. (2021), which is based on matching and weighting methods to refine the comparison between treated and control units. Differently from other approaches commonly applied to difference-in-differences analyses (De Chaisemartin and d’Haultfoeuille, 2018, Callaway and Sant’Anna, 2021, Goodman-Bacon, 2021), this method better fits our setting because it foresees that units may switch in and out of the treatment. We perform matching on the basis of the lagged value of the dependent variable, the level of violence and the region to which the province belongs. The standard errors are bootstrapped 1000 times. Fig. B.1 (left) illustrates that most matched control units present similar history as the treatment units. Fig. B.1 (right) shows the matching estimates of the effects of femicides on help-seeking behavior. As in our main results, the point estimate is positive and statistically significant in period t+1 and not different from zero elsewhere.

Finally, we conduct an additional falsification exercises in the spirit of placebo tests to strengthen the causal mechanism. To address the possibility that the main results are driven by spurious correlations or seasonal trends, we propose a randomization test based on future femicides, i.e. those occurring in the same week and subsequent year. The coefficients related to this exercise, reported in Fig. B.2, are small in magnitude and never statistically significant at any conventional level, thus alleviating concerns over the existence of a spurious relationship.

## Appendix C Google search and news coverage data

### C.1 Google searches

Starting with the seminal contribution of Choi and Varian (2012), Google search data proved useful to proxy a variety of economic and social phenomena.

Google Trends (https://trends.google.com/trends/) provides access to the search requests made to the Google search engine by its users. It contains a random sample representative of all queries that Google handles daily. Search results are normalized to the time and location of a query. Each data point is divided by the total searches by time range and geography (in our case, week and region), to obtain relative popularity. Depending on the type of access, the resulting numbers are (i) scaled on a range of 0 to 100 based on a query’s proportion to all searches on all queries; or (ii) multiplied by 10 million to reduce the number of decimals.

We exploit the latter access and construct a time series of weekly Google searches for each victim’s name over the period 2015–2019 at the national level.

For each victim, we consider Google searches within a time window that runs from 8 weeks before to 8 weeks after the femicide. Note that in case of homonyms, Google searches could be positive even before the date of the femicide. To account for such (few) cases, we de-mean each series by rescaling the after-femicide intensity of Google searches by the before-femicide intensity. We then rank all post-femicide searches and define the dummy variable *Most Searched* as equal to 1 if the value of the Google Search index is in the highest quartile of the sample distribution, and 0 otherwise.

### C.2 News coverage data

News coverage data are collected from GDELT, the *Global Database of Events, Language and Tone* (https://www.gdeltproject.org/). GDELT is an open, big data platform of meta-information extracted from broadcast, print, and web news collected worldwide and translated into English from over 65 different languages. GDELT consists of several datasets, the most comprehensive one being the GDELT Global Knowledge Graph (GDELT-GKG), which analyses over 88 million articles a year and more than 150,000 news outlets.

Each item (news) in the GDELT-GKG data provides information on people, locations and organizations mentioned in the text of the article and retrieves counts, quotes, images and themes using several popular topical taxonomies. We exploit these taxonomies to retrieve all news related to gender violence, which resulted in the following meta-tag: ‘gender violence’, ‘rape’, ‘youth and gender based violence’, ‘domestic violence’, ‘rape and sexual violence’.^33^

We then restrict our sample to all news from stable sources in Italy over the period 2016–2019. Note that GDELT-GKG is available also in 2015, but since this is the year of deployment of the GDELT infrastructure, the number of articles parsed is not constant over time. For this reason we exclude 2015. Such restriction allows us to increase the stability and coherence in the outlets parsed.

We define a daily measure of news coverage of gender-based violence – $GD_{d}$ – that is equal to the share of tagged news over the total number of news in a given day d. Since our objective is to identify femicides that receive most coverage, we devise an ad-hoc procedure to statistically detect temporary increases in media coverage of news related to gender-based violence.^34^

This procedure is based on pre-post rolling windows comparisons. More specifically, we define $d_{0}$ as the beginning of the observable $GD_{d}$ daily time series. We keep observations between $d_{0}$ and $d_{0}+30$, and define a Post dummy for the 15-days sub-period between $d_{0}+15$ and $d_{0}+30$. We use these observations to estimate the regression $GD_{d}=\gamma_{0}+\gamma_{1}Post_{d}+\epsilon_{d}$. After storing the estimated coefficient $\gamma_{1}$ and its standard error, we roll forward the time window by one day and re-estimate the same regression. We iterate this procedure until the end of the observable time series. The result of this iterative procedure is a series of estimated coefficients and their corresponding standard errors. Importantly, for each day d there are 15 coefficient estimates, one for each position of day d in the rolling Post windows.

We then compute, for each day, the fraction of iterations in which the $\gamma_{1}$ coefficient is positive and statistically significant. These shares are used to identify the increase in coverage and the subsequent decrease. Intuitively, as the time series starts to trend up, post periods are more likely to be associated with positive and statistically significant $\gamma_{1}$ coefficients, and so the share for each day increases with the trend. The opposite happens when the series starts to trend down, with a lower likelihood of positive and significant $\gamma_{1}$ coefficients, and decreasing shares. We define the beginning of a period of increasing coverage as the first day with a positive share of significant $\gamma_{1}$ coefficients preceded by a day with a share equal to zero. We identify the peak of the temporary increase as the first day with a share equal to 1. Finally, we define the *Most Covered* dummy variable which is equal to 1 for femicides occurring between the beginning and peak of a period of increasing coverage, and 0 otherwise.

## Appendix D The effect of a femicide news

An IPV survivor takes action against violence if condition (2) holds, i.e. $\mu_{i,t}>{\overset{ˆ}{\mu }}_{i,t}$, where

(5) $${\overset{ˆ}{\mu }}_{i,t}=\frac{p_{i,t}}{q_{i,t}}V_{i,t}-\frac{1-p_{i,t}}{q_{i,t}}B_{i,t}-\rho v_{i,t-1}$$

Consider the case in which femicide news affect all beliefs and payoffs:

$$p=p\left(n\right),q=q\left(n\right),B=B\left(n\right),V=V\left(n\right),\mu =\mu \left(n\right),\rho =\rho \left(n\right)$$

Seeking for help is more likely after a femicide news if $\mu^{\prime }>{\overset{ˆ}{\mu }}^{\prime }$, i.e. if

$$\mu^{\prime }>\frac{1}{q}\left[\left(B+V\right)p^{\prime }+pV^{\prime }-\left(1-p\right)B^{\prime }+\frac{\left(1-p\right)B-pV}{q}q^{\prime }\right]-v_{t-1}\rho^{\prime }$$

The first three terms in the square bracket describe the change in the expected payoff associated to taking action after a femicide news is released. In the simple case considered in the text, where $V^{\prime }=B^{\prime }=0$, the effect is captured by the change in the beliefs p about the probability of violence as retaliation. The remaining terms describe the effect of the femicide news on not taking action. In the main text, $q^{\prime }=\rho^{\prime }=0$, hence this effect boils down to the change in μ.

## Footnotes

1 For an overview on policies and campaigns implemented worldwide, see the UN Women’s Global Database on Violence Against Women, available at https://evaw-global-database.unwomen.org/en.

2 See DellaVigna and La Ferrara (2015) for a comprehensive review of the economic impact of media exposure.

3 In Italy, femicides are usually reported by national media on the day the news are released. Although local newspapers may report more details, the variables we consider in the analysis (date, place, identity of the victim and of her killer – if known –, motive and the way she was murdered) are reported on national media. Thus, femicides are likely to be correctly reported and classified in our data. When comparing the aggregate number of murders with female victims registered by ISTAT official statistics and the events in our sample, we do not observe substantial differences. For instance, in 2018 ISTAT reports 133 episodes of female homicides, our data reports 115 records. Note that, while ISTAT reports counts for all murders, we consider only gender-based ones.

4 In 2019, there were 302 AVCs in Italy. They operate at the local level and offer services such as individual or group counseling (92.2%), legal support (93.8%), job search support (80.5%), parenting support (62.3%).

5 Year 2015 is missing because GDELT data covers a scant number of news outlets. For details on the construction of the Google Searches and news coverage indexes see Appendix C.

6 In the last decades Italy has experienced a decreasing trend in intentional homicides and is nowadays one of the countries with the lowest homicide rates in the world. With about 0.5 homicides per 100,000 inhabitants, it ranks well below the OECD average of 2.6 and the USA average of 6.0 per 100,000 inhabitants (OECD, 2022).

7 We define femicides as the intentional murders of women because of their gender. This notion is not undisputed, as broader definitions can include any intentional murder of women and girls (see, e.g., WHO, 2012).

8 Other causes of death include firearm, stabbing, poisoning and death by gas.

9 A notable exception occurs when the offender is caught in the act by the police. The so-called “Codice Rosso” bill, passed in July 2019, has introduced new measures against gender-based and domestic violence, among which the prospect of nearly immediate (i.e. within three days) arrest for threats, violence and stalking. Our results are invariant to excluding the post-July 2019 period.

10 Information on the reasons for calling and on the identity of the user or offender are only available since 2018, at the national level and yearly. Hence they cannot be used in our empirical analysis.

11 The fraction of third parties reporting violence has increased substantially during the Covid-19 pandemic and the associated lockdown measures (ISTAT, 2021b). As year 2020 is an outlier, it is not considered in our analysis.

12 The helpline receives most calls during the traditional business hours (64% between 9 AM and 17 PM) and only about 20% over weekends. This suggests that help-seekers reach out especially when abusers are more likely to be out of the house for work. Nearly all victims are female (97%) and in more than 45% of the cases they are younger than 44. The large majority is either married or divorced and less than 30% is unmarried. Almost 40% of callers are employed, 20% are unemployed, 12% are housewives, 11% are retiree and 4% are students. Educational attainment is homogeneously distributed across middle-school, high-school and university level. Note that callers do not always answer to questions related to their demographics. Missing entries range from 5% to over 50%.

13 Data in panel (b) of Fig. 3 refer to the total yearly criminal records and charges, where the victim is a woman, for the following crimes: injuries, blows, sexual violence, stalking, attempted homicide, kidnapping. They are used for descriptive purpose since available only at region-year level up to 2016. The empirical analysis in Section 5 exploits a subset of crimes (i.e. domestic abuse and maltreatments) that are specifically defined by the Police as crimes associated to gender-based violence.

14 To ease the comparison with the indicator referred to police reports, which is not available on a weekly basis, the two maps display monthly averages.

15 The model can potentially describe also the decision problem of an altruistic person (e.g., a relative or a friend) who cares about the well being of the IPV survivor. For expositional simplicity, in the text we refer to the IPV survivor only.

16 Unless confusion can arise, we omit the subscripts i and t from probabilities and payoffs.

17 This outcome corresponds to the hope that “the abusive partner will change”, which the literature describes as a possible motivation for not leaving the abusive partner (Heise and Garcia-Moreno, 2002).

18 The process generating $\mu_{i,t}$ is assumed to be stationary. A richer theoretical model could explicitly consider the choices available to the abuser and consider an equilibrium in which both the IPV survivor and the abuser make strategic choices (see, e.g., Bloch and Rao, 2002, Bowlus and Seitz, 2006, Cheng and Hsiaw, 2022). This would allow to study, for instance, how the abuser’s behavior responds the femicide news, or how it responds to the expectation that the femicide news makes a victim more likely to report. We opt for a different approach, which emphasizes the role of the survivor’s past history of violence in affecting the expected trajectory of violence in case no action is taken. Despite its simplicity, this model delivers intuitive results about the main determinants and trade-offs affecting a survivor’s choice of seeking for help, which is our main empirical focus.

19 Heise and Garcia-Moreno (2002, p.95) observe that women leave their abusive partners permanently “when the violence becomes severe enough to trigger the realization that the partner is not going to change, or when the situation starts noticeably to affect the children”. In terms of our model, this corresponds to large values of $q_{i}$ and $v_{i,t}$.

20 See Appendix D for a model in which the femicide news affects all payoffs and beliefs.

21 Robustness exercises based on different values of π provide similar conclusions.

22 This procedure is relevant in staggered difference-in-differences (DiD) designs where the treatment effects vary over time. When this is the case, the DiD estimates a variance-weighted average of treatment effect parameters with “negative weights” (see, e.g., Sun and Abraham, 2021, De Chaisemartin and d’Haultfoeuille, 2020). For a survey on the recent DiD literature, see De Chaisemartin and d’Haultfoeuille (2022).

23 Here, the dummies associated to π=−3 and τ=2 include all periods prior to t−3 (up to t−4) and those after t+2 (up to t+6), respectively.

24 It is reasonable to think that at least some close family members who report violence are themselves indirectly victims of violence (e.g., a child that reports violence on her mother). However, in our data calls made by family or friends or by professionals are around 5% of the total calls (see Table 1). Note also that, by focusing on pre-Covid years, the evidence on third party reporting uncovered during Covid-related stay-at-home orders (Ivandic et al., 2021, Miller et al., 2022), may not directly apply.

25 Extending the treatment at the regional level yields the same results.

26 Results hold when we exclude the weeks coinciding with the International Women’s Day (March 8) and the International Day for the Elimination of Violence against Women (November 25).

27 We find no detectable effect of other characteristics of the femicide, such as, e.g., the existence of a previous history of violence before the femicide occurred. This null result could be due to certain features of the femicide being truly not relevant for the callers, or to lack of power.

28 The fact that IPV survivors decide to call the helpline does not necessarily imply that they will immediately leave their abusers. The way out of IPV is an uncertain and long process that can take time. Indeed, the 1522 statistics describe a complex path, where only three quarters of the calls are made by people that call for the first time. See the ISTAT portal at http://dati-violenzadonne.istat.it/.

29 See the ISTAT portal at http://dati-violenzadonne.istat.it/.

30 See Eurostat at https://ec.europa.eu/eurostat/web/products-eurostat-news/-/ddn-20210224-1.
